# Supplementary material for: The Interaction Between Orientin and the Spike of SARS-CoV-2: An In Silico and Experimental Approach
Source: Viruses. 2025 Dec 31;18(1):61. doi: 10.3390/v18010061 (PMC12846643; doi:10.3390/v18010061)
Supplement: Supplementary file 1 [file viruses-18-00061-s001.zip › viruses-4013178-supplementary.pdf]

Interaction Between Orientin and Spike of SARS-CoV-2: An *in silico* and Experimental Approach

Supplementary Figures

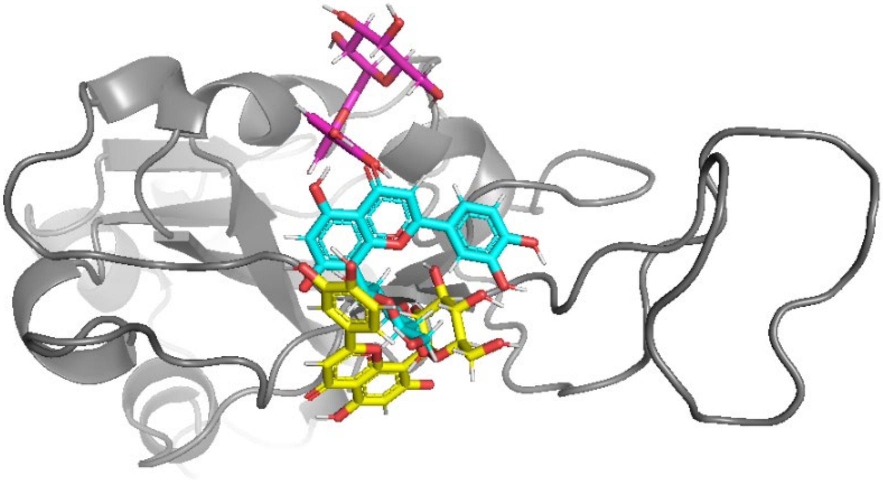

Figure S1. Overlay of the three best modes of Orientin generated from docking experiments. Orientin's Best Binding Mode Number 2 (from the molecular docking using 6M0J – without ACE2 - pdb file) is colored in cyan, Best Biding Mode Number 15 (from the molecular docking using 7BZ5 pdb file) is colored in magenta, and Best Biding Mode Number 4 (from the molecular docking using 6M0J – with ACE2 - pdb file) is colored in yellow.

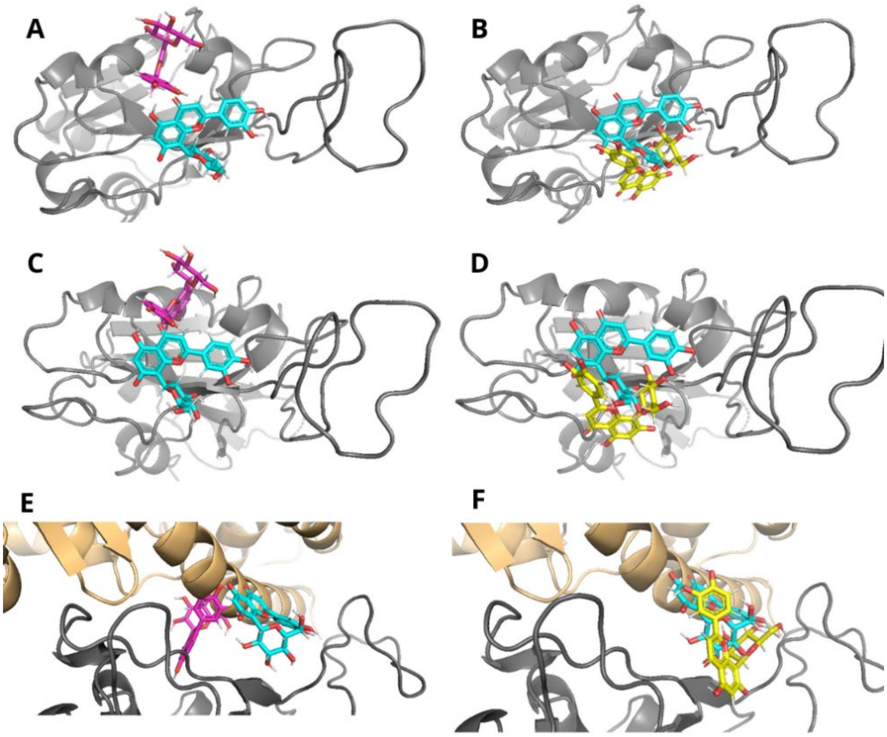

Figure S2. Overlay of the best modes of Orientin generated from docking experiments with RBD without ACE2 (A and B), RBD without antibody and ACE2 (C and D) and RBD with ACE2 (D and E). Orientin's Best Binding Mode Number 2 (from the molecular docking using 6M0J – without ACE2 - pdb file) is colored in cyan, Best Biding Mode Number 15 (from the molecular docking using 7BZ5 pdb file) is colored in magenta, and Best Biding Mode Number 4 (from the molecular docking using 6M0J – with ACE2 - pdb file) is colored in yellow.

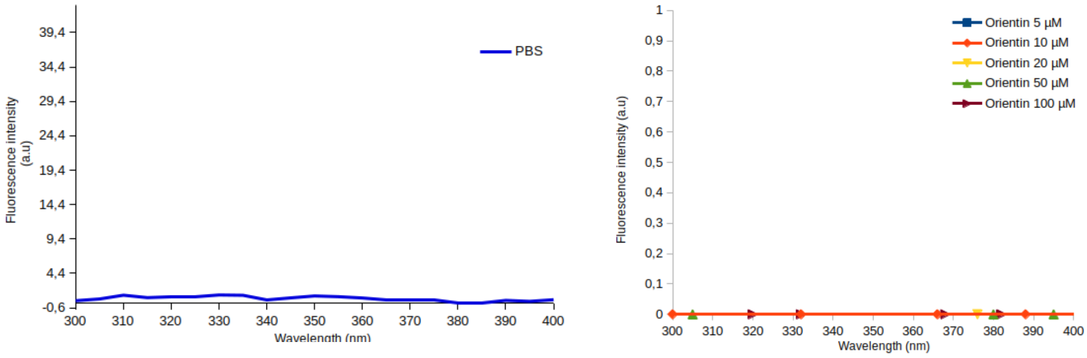

Figure S3. Emission spectrum of fluorescence from PBS and Orientin. PBS does not show a fluorescence emission peak between 300-420 nm (A). Different concentrations of Orientin in PBS indicate that the compound does not emit fluorescence at the same wavelength as Glycoprotein S (B).
